# Supplementary material for: Bacterial communities of the psyllid pest Bactericera cockerelli (Hemiptera: Triozidae) Central haplotype of tomato crops cultivated at different locations of Mexico
Source: PeerJ. 2023 Nov 3;11:e16347. doi: 10.7717/peerj.16347 (PMC10629388; doi:10.7717/peerj.16347)
Supplement: Supplemental Information 1 — Most conserved parts are depicted in red. The figure was generated using MultAlin software. *one single-nucleotide polymorphism (SNP) that exists between the Western and Central haplotypes in a 500-bp fragment of the mtCOI gene. [file peerj-11-16347-s001.pdf]

```
                  1 *      10        20        30        40        50        60        70        80        90        100       110       120       130
                  |--------+---------+---------+---------+---------+---------+---------+---------+---------+---------+---------+---------+---------|
Sinaloa           TCATATATTGACAGTAGGTATAGATGTTGATTCTCGTGCCTATTTCACTTCCGCAACTATAATTATTGCTGTCCCTACAGGAATTAAAATTTTTAGTTGATTAGCAACTATTTATGGGATAAAAATATAT
Queretaro         TCATATATTGACAGTAGGTATAGATGTTGATTCTCGTGCCTATTTCACTTCCGCAACTATAATTATTGCTGTCCCTACAGGAATTAAAATTTTTAGTTGATTAGCAACTATTTATGGGATAAAAATATAT
Coahuila          TCATATATTGACAGTAGGTATAGATGTTGATTCTCGTGCCTATTTCACTTCCGCAACTATAATTATTGCTGTCCCTACAGGAATTAAAATTTTTAGTTGATTAGCAACTATTTATGGGATAAAAATATAT
Nuevo Leon        TCATATATTTACAGTAGGTATAGATGTTGATTCTCGTGCCTATTTCACTTCCGCAACTATAATTATTGCTGTCCCTACAGGAATTAAAATTTTTAGTTGATTAGCAACTATTTATGGGATAAAAATATAT
FJ175374.1Central TCATATATTTACAGTAGGTATAGATGTTGATTCTCGTGCCTATTTCACTTCCGCAACTATAATTATTGCTGTCCCTACAGGAATTAAAATTTTTAGTTGATTAGCAACTATTTATGGGATAAAAATATAT
EF372597.1Central TCATATATTTACAGTAGGTATAGATGTTGATTCTCGTGCCTATTTCACTTCCGCAACTATAATTATTGCTGTCCCTACAGGAATTAAAATTTTTAGTTGATTAGCAACTATTTATGGGATAAAAATATAT
JQ708095.1Western TCACATATTTACAGTAGGTATAGATGTTGATTCTCGTGCCTATTTCACTTCCGCAACTATAATTATTGCTGTCCCTACAGGAATTAAAATTTTTAGTTGATTAGCAACTATTTATGGGATAAAAATATAT
AY971885.1Western TCACATATTTACAGTAGGTATAGATGTTGATTCTCGTGCCTATTTCACTTCCGCAACTATAATTATTGCTGTCCCTACAGGAATTAAAATTTTTAGTTGATTAGCAACTATTTATGGGATAAAAATATAT
Consensus         TCAcATATTtACAGTAGGTATAGATGTTGATTCTCGTGCCTATTTCACTTCCGCAACTATAATTATTGCTGTCCCTACAGGAATTAAAATTTTTAGTTGATTAGCAACTATTTATGGGATAAAAATATAT

                  131      140       150       160       170       180       190       200       210       220       230       240       250       260
                  |--------+---------+---------+---------+---------+---------+---------+---------+---------+---------+---------+---------+---------|
Sinaloa           TTTTCTCCAAGTATTATTTGATCTCTAGGATTCATTTTCCTGTTTACACTGGGAGGTTTAACAGGTGTAATTTTAGCAAATTCTTCAATTGACATTATTTTACATGACACATACTATGTAGTAGCACATT
Queretaro         TTTTCTCCAAGTATTATTTGATCTCTAGGATTCATTTTCCTGTTTACACTGGGAGGTTTAACAGGTGTAATTTTAGCAAATTCTTCAATTGACATTATTTTACATGACACATACTATGTAGTAGCACATT
Coahuila          TTTTCTCCAAGTATTATTTGATCTCTAGGATTCATTTTCCTGTTTACACTGGGAGGTTTAACAGGTGTAATTTTAGCAAATTCTTCAATTGACATTATTTTACATGACACATACTATGTAGTAGCACATT
Nuevo Leon        TTTTCTCCAAGTATTATTTGATCTCTAGGATTCATTTTCCTGTTTACACTGGGAGGTTTAACAGGTGTAATTTTAGCAAATTCTTCAATTGACATTATTTTACATGACACATACTATGTAGTAGCACATT
FJ175374.1Central TTTTCTCCAAGTATTATTTGATCTCTAGGATTCATTTTCCTGTTTACACTGGGAGGTTTAACAGGTGTAATTTTAGCAAATTCTTCAATTGACATTATTTTACATGACACATACTATGTAGTAGCACATT
EF372597.1Central TTTTCTCCAAGTATTATTTGATCTCTAGGATTCATTTTCCTGTTTACACTGGGAGGTTTAACAGGTGTAATTTTAGCAAATTCTTCAATTGACATTATTTTACATGACACATACTATGTAGTAGCACATT
JQ708095.1Western TTTTCTCCAAGTATTATTTGATCTCTAGGATTCATTTTCCTGTTTACACTGGGAGGTTTAACAGGTGTAATTTTAGCAAATTCTTCAATTGACATTATTTTACATGACACATACTATGTAGTAGCACATT
AY971885.1Western TTTTCTCCAAGTATTATTTGATCTCTAGGATTCATTTTCCTGTTTACACTGGGAGGTTTAACAGGTGTAATTTTAGCAAATTCTTCAATTGACATTATTTTACATGACACATACTATGTAGTAGCACATT
Consensus         TTTTCTCCAAGTATTATTTGATCTCTAGGATTCATTTTCCTGTTTACACTGGGAGGTTTAACAGGTGTAATTTTAGCAAATTCTTCAATTGACATTATTTTACATGACACATACTATGTAGTAGCACATT

                  261      270       280       290       300       310       320       330       340       350       360       370       380       390
                  |--------+---------+---------+---------+---------+---------+---------+---------+---------+---------+---------+---------+---------|
Sinaloa           TCCATTATGTTCTATCTATAGGGGCTGTATTTGCAATTATTGCTAGATTTATTAATTGATACCCTTTAATAACAGGAGTAATTATAAATAAAACTTTATTAAAAACACAATTTATTAGTACTTTTATTGG
Queretaro         TCCATTATGTTCTATCTATAGGGGCTGTATTTGCAATTATTGCTAGATTTATTAATTGATACCCTTTAATAACAGGAGTAATTATAAATAAAACTTTATTAAAAACACAATTTATTAGTACTTTTATTGG
Coahuila          TCCATTATGTTCTATCTATAGGGGCTGTATTTGCAATTATTGCTAGATTTATTAATTGATACCCTTTAATAACAGGAGTAATTATAAATAAAACTTTATTAAAAACACAATTTATTAGTACTTTTATTGG
Nuevo Leon        TCCATTATGTTCTATCTATAGGGGCTGTATTTGCAATTATTGCTAGATTTATTAATTGATACCCTTTAATAACAGGAGTAATTATAAATAAAACTTTATTAAAAACACAATTTATTAGTACTTTTATTGG
FJ175374.1Central TCCATTATGTTCTATCTATAGGGGCTGTATTTGCAATTATTGCTAGATTTATTAATTGATACCCTTTAATAACAGGAGTAATTATAAATAAAACTTTATTAAAAACACAATTTATTAGTACTTTTATTGG
EF372597.1Central TCCATTATGTTCTATCTATAGGGGCTGTATTTGCAATTATTGCTAGATTTATTAATTGATACCCTTTAATAACAGGAGTAATTATAAATAAAACTTTATTAAAAACACAATTTATTAGTACTTTTATTGG
JQ708095.1Western TCCATTATGTTCTATCTATAGGGGCTGTATTTGCAATTATTGCTAGATTTATTAATTGATACCCTTTAATAACAGGAGTAATTATAAATAAAACTTTATTAAAAACACAATTTATTAGTACTTTTATTGG
AY971885.1Western TCCATTATGTTCTATCTATAGGGGCTGTATTTGCAATTATTGCTAGATTTATTAATTGATACCCTTTAATAACAGGAGTAATTATAAATAAAACTTTATTAAAAACACAATTTATTAGTACTTTTATTGG
Consensus         TCCATTATGTTCTATCTATAGGGGCTGTATTTGCAATTATTGCTAGATTTATTAATTGATACCCTTTAATAACAGGAGTAATTATAAATAAAACTTTATTAAAAACACAATTTATTAGTACTTTTATTGG

                  391      400       410       420       430       440       450  455
                  |--------+---------+---------+---------+---------+---------+----|
Sinaloa           TGTTAACCTTACTTTTTTCCCCCAACATTTCTTAGGACTCATAGGAATACCACGACGTTACTCAA
Queretaro         TGTTAACCTTACTTTTTTCCCCCAACATTTCTTAGGACTCATAGGAATACCACGACGTTACTCAA
Coahuila          TGTTAACCTTACTTTTTTCCCCCAACATTTCTTAGGACTCATAGGAATACCACGACGTTACTCAA
Nuevo Leon        TGTTAACCTTACTTTTTTCCCCCAACATTTCTTAGGACTCATAGGAATACCACGACGTTACTC
FJ175374.1Central TGTTAACCTTACTTTTTTCCCCCAACATTTCTTAGGACTCATAGGAATACCACGACGTTACTC
EF372597.1Central TGTTAACCTTACTTTTTTCCCCCAACATTTCTTAGGACTCATAGGAATACCACGACGTTACTC
JQ708095.1Western TGTTAACCTTACTTTTTTCCCCCAACATTTCTTAGGACTCATAGGAATACCACGACGTTACTC
AY971885.1Western G
Consensus         tgttaaccttacttttttcccccaacatttcttaggactcataggaataccacgacgttactc..
```
