# Supplementary figures and images for: Bacterial communities of the psyllid pest Bactericera cockerelli (Hemiptera: Triozidae) Central haplotype of tomato crops cultivated at different locations of Mexico
Source: PeerJ. 2023 Nov 3;11:e16347. doi: 10.7717/peerj.16347 (PMC10629388; doi:10.7717/peerj.16347)

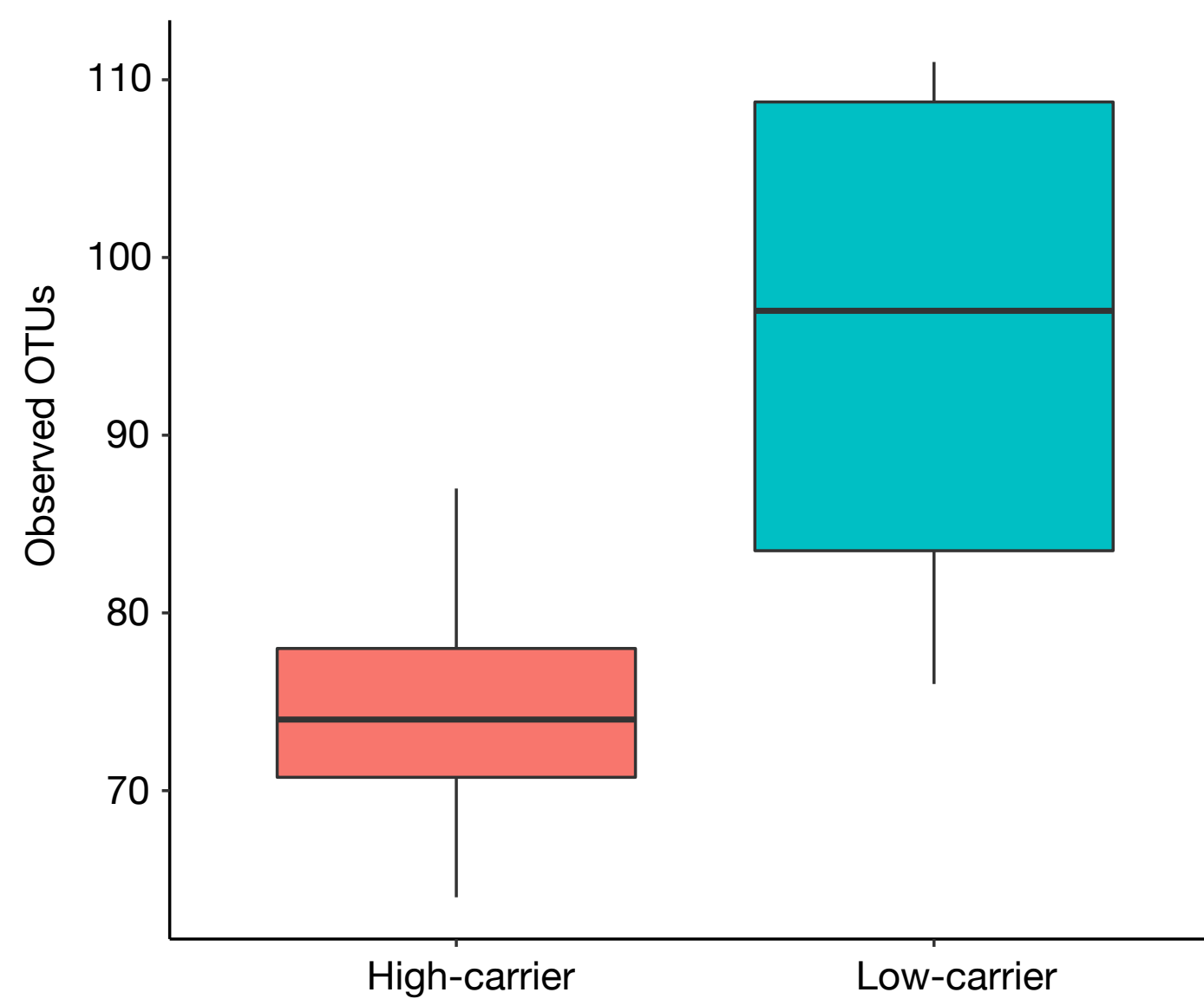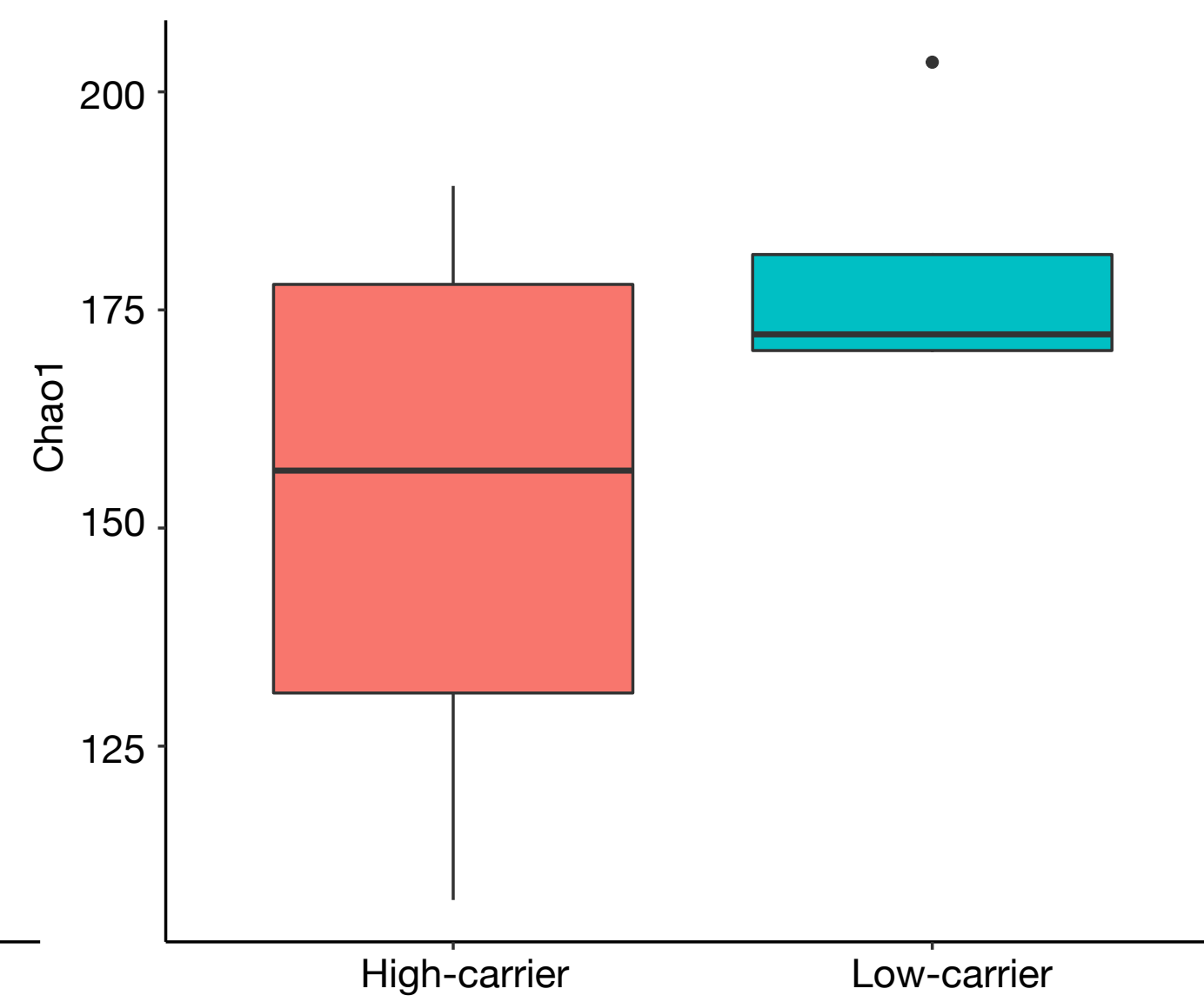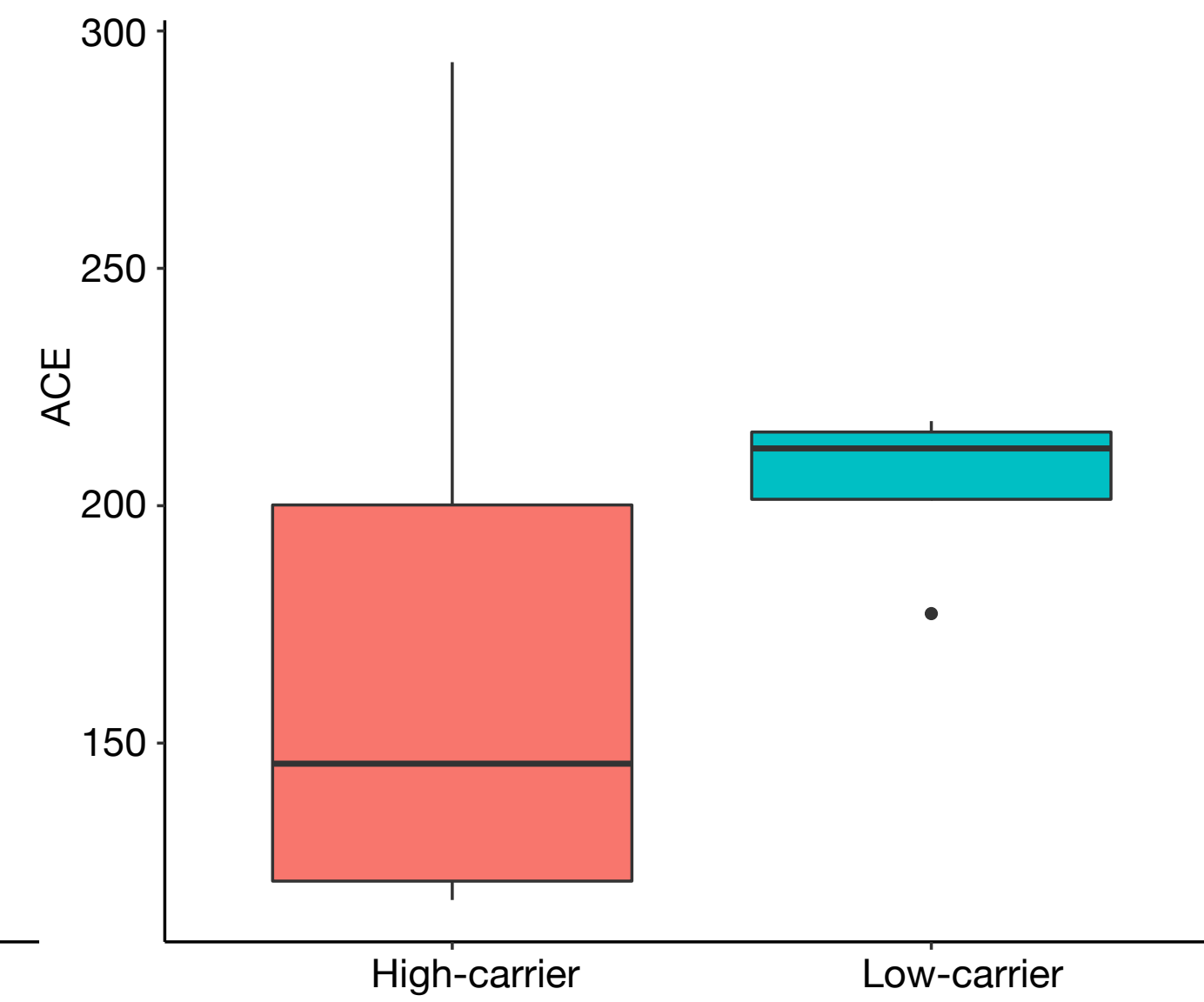

High-carrier  
Low-carrier

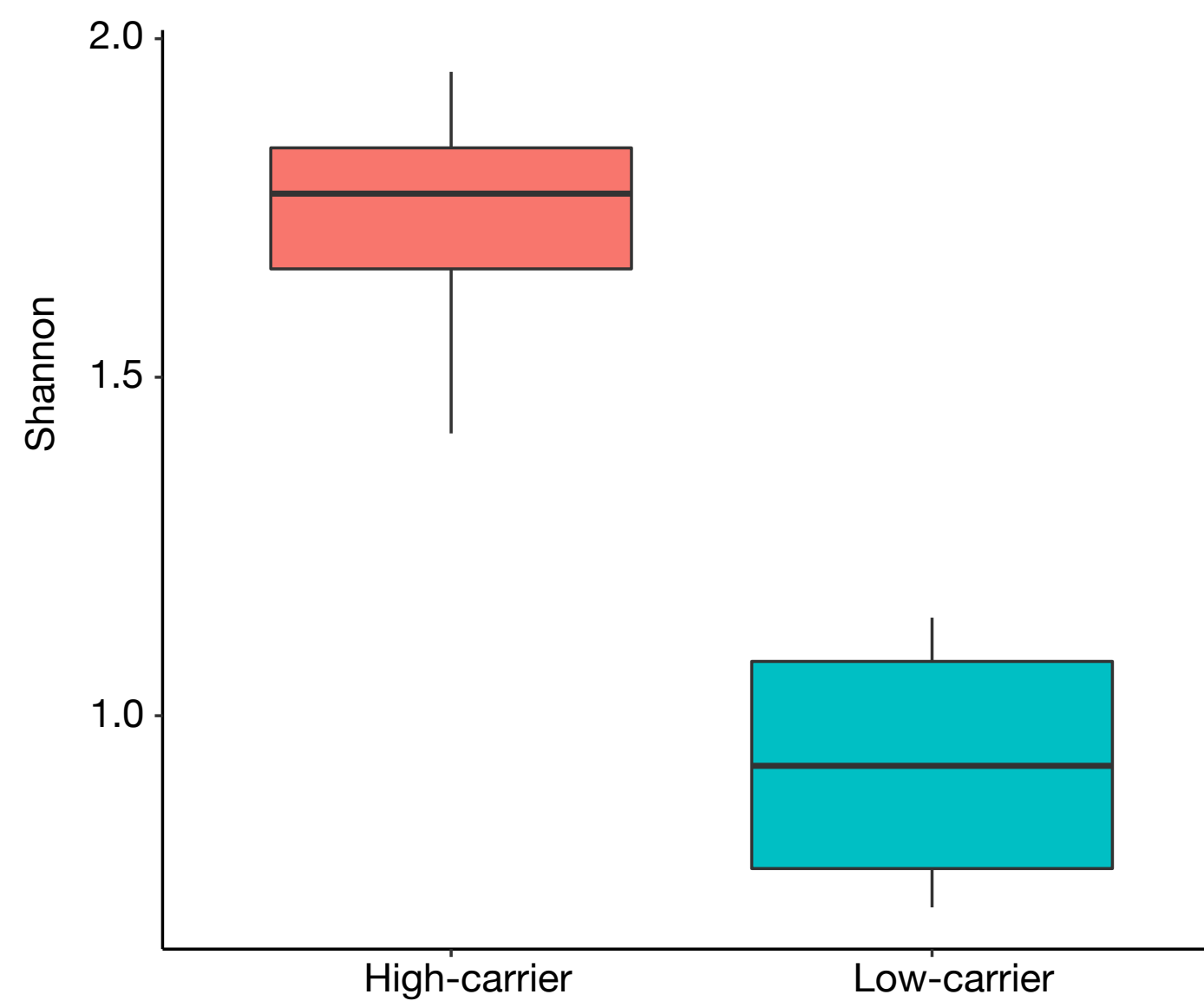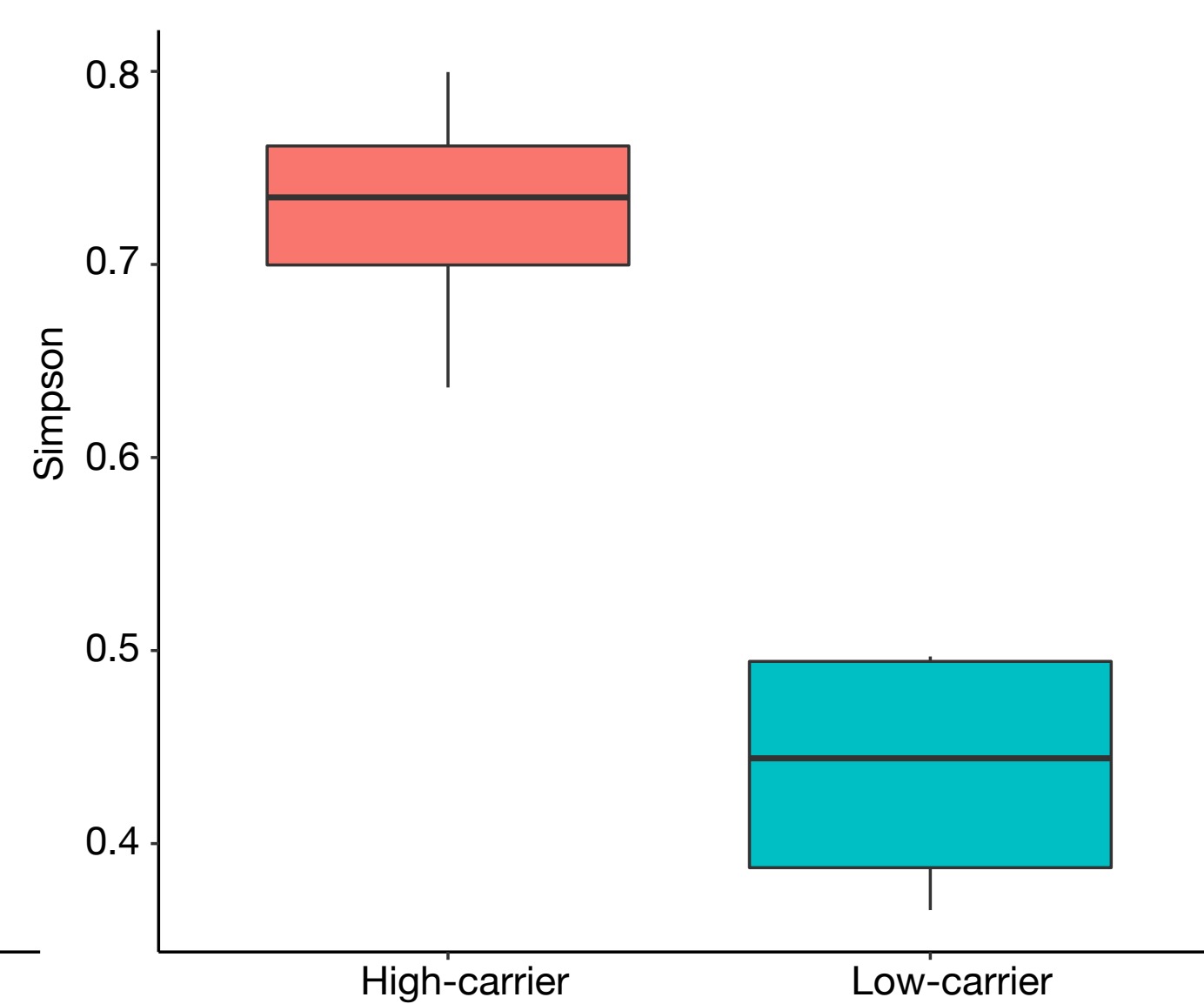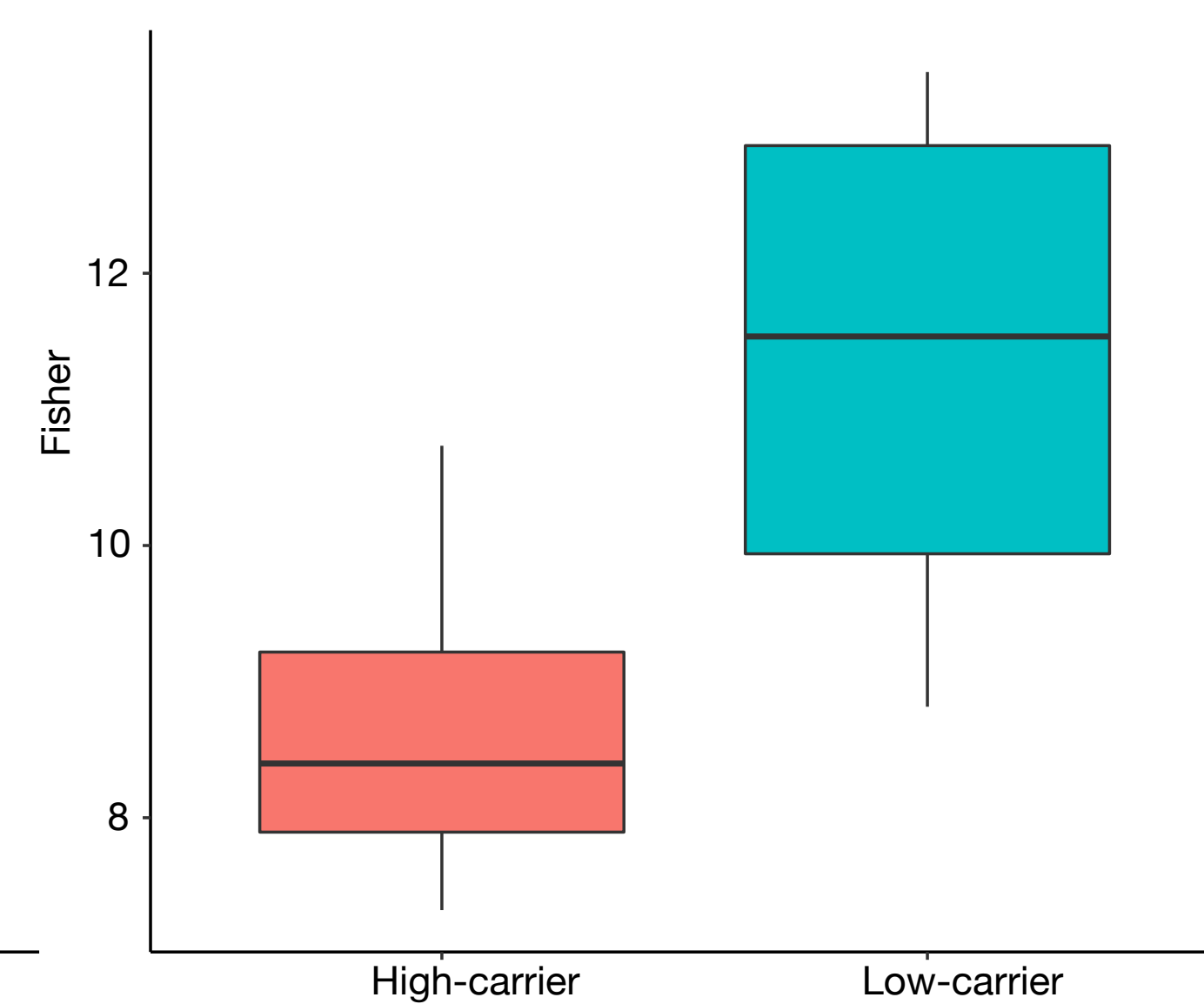

Supplement: Supplemental Information 2 — (a) OTUs, (b) Chao1, (c) ACE, (d) Shannon, (e) Simpson, and (f) Fisher. Boxes represent the interquartile range, and the horizontal line inside the box defines the median. [file peerj-11-16347-s002.pdf]
